# Transcriptional silencing of the *Dickkopfs-3* (*Dkk-3*) gene by CpG hypermethylation in acute lymphoblastic leukaemia

**DOI:** 10.1038/sj.bjc.6602008

**Published:** 2004-06-29

**Authors:** J Roman-Gomez, A Jimenez-Velasco, X Agirre, J A Castillejo, G Navarro, M Barrios, E J Andreu, F Prosper, A Heiniger, A Torres

**Affiliations:** 1 Hematology Department, Molecular Biology Unit, Reina Sofia Hospital, 14004 Cordoba, Spain; 2 Hematology Department, Molecular Biology Unit, Carlos Haya Hospital, Malaga, Spain; 3 Hematology Department, Cellular Therapy Area, Clinica Universitaria/School of Medicine, Foundation for Applied Medical Research, University of Navarra, Pamplona, Spain

**Keywords:** acute lymphoblastic leukaemia, CpG island, methylation, *Dkk*-3

## Abstract

*Dkk-3* is a newly characterised mortalisation-related gene and an antagonist of the Wnt oncogenic signalling pathway whose expression is decreased in a variety of cancer cell lines, suggesting that the *Dkk-3* gene, located at chromosome 11p15.1, functions as a tumour suppressor gene. Although 11p15 is a ‘hot spot’ for methylation in acute lymphoblastic leukaemia (ALL), the role of *Dkk-3* abnormalities has never been evaluated in this disease. We analysed CpG island methylation of the *Dkk-3* promoter in six ALL cell lines and 183 ALL patients. We observed *Dkk-3* hypermethylation in all cell lines and in cells from 33% (60/183) of ALL patients. Moreover, *Dkk-3* methylation was associated with decreased *Dkk-3* mRNA expression and this expression was restored after exposure to the demethylating agent 5-AzaC. Clinical features did not differ between hypermethylated and unmethylated patients. Estimated disease-free survival (DFS) and overall survival at 10 and 11 years, respectively, were 49.8 and 45.6% for normal patients and 10.5 and 15.1% for hypermethylated patients (*P*=0.001 and 0.09). Multivariate analysis demonstrated that *Dkk-3* methylation was an independent prognostic factor predicting DFS (*P*=0.0009). Our data suggest that *Dkk-3* methylation occurs at an early stage in ALL pathogenesis and probably influences the clinical behaviour of the disease.

DNA methylation is an essential mechanism for the regulation of gene expression in mammalian cells (Bird, 2002). Methylation occurs at cytosine residues within CpG dinucleotides and many genes are enriched with these dinucleotides in their promoters. These regions are known as CpG islands and are generally nonmethylated, a condition that allows genes to be transcriptionally competent. Methylation of CpG islands within gene promoters leads to transcriptional silencing through recruitment of methyl-CpG binding protein and histone deacetylases (Jones and Baylin, 2002). Hence, identification of the methylation patterns of CpG islands in mammalian cells is important for understanding normal and pathological gene expression. Several reports have shown that abnormal hypermethylation of CpG islands may contribute significantly to the pathogenesis of human leukaemias. Hypermethylation of promoters associated with genes involved in the control of the principal late-G1 cell-cycle checkpoint, the apoptotic programme and the cell–cell adhesion is detected with increased frequency in acute lymphoblastic leukaemia (ALL) (Roman-Gomez *et al*, 2003). This mechanism provides an alternative route to gene mutation of cancer-related genes. Although genomewide methylation changes in CpG islands are early and frequent events in ALL, it is also evident that some of these changes occur preferentially in specific chromosome regions. In this context, 11p15 region seems to represent a ‘hot spot’ for methylation in ALL. In fact, epigenetic inactivation of *Calcitonin* (11p15.1–15.2), *p57* (11p15.5) and *Myf-3* (11p15.4) genes are frequently observed in both childhood and adult ALL (Roman-Gomez *et al*, 2001; Garcia-Manero *et al*, 2002; Li *et al*, 2002). Interestingly, *Calcitonin* gene is methylated in a large proportion of ALL patients with dismal prognosis. However, this gene is generally considered not to be causally related to the malignant process suggesting that its methylation is a surrogate for the methylation of another potential tumour suppressor gene located in its vicinity.

The human *Dickkopfs-3* (*Dkk-3*) gene, located on chromosome 11p15.1 is a recently found mortalisation-related gene expression of which is largely attenuated in many immortalised and tumour-derived cell lines (Tsuji *et al*, 2000). Furthermore, by transfection experiments, it has been determined that *Dkk-3* possesses an antiproliferative activity against tumour cells, suggesting that *Dkk-3* may function as a tumour suppressor (Tsuji *et al*, 2001).

Recent studies have shown that some Dkks play a role in a wide range of normal and pathological developmental processes, including cancer, and that their effects seem to be mediated by their ability to antagonise Wnt signalling (Taipale and Beachy, 2001). Wnt signalling pathway has profound effects in normal haematopoiesis inducing bone marrow cells to develop into a variety of different lymphoid cell types and also augmenting repopulating capacity and primitive hematopoietic development of human blood stem cell *in vivo* (Brandon *et al*, 2000). In addition, expression of Wnt-16 following a chromosomal translocation has been reported in preB-cell ALL (McWhirter *et al*, 1999) and Wnt5a hemizygous mice develop myeloid leukaemias and B-cell lymphomas that are clonal in origin suggesting a role for Wnts in leukaemogenesis. However, little is known about the potential role of Wnt antagonists in lymphoid neoplasia.

In this paper, we demonstrate that the putative tumour suppressor *Dkk-3* gene is silenced by methylation in ALL and that this epigenetic event confers poor prognosis in this group of patients.

## MATERIALS AND METHODS

### Cell lines and samples

Four human precursor-B ALL (BV173, TOM-1, MY and NALM-20) and two T-cell leukaemia (MOLT-3 and MOLT-4) cell lines were obtained from the American Type Culture Collection (Manassas, VA, USA). The cells were cultured in the appropriate medium until harvested for extraction of DNA and RNA using standard procedures. Bone marrow samples obtained by aspiration were collected after an acquisition of informed consent from 183 consecutive patients (106 male; 77 female) who were diagnosed with *de novo* ALL between January 1989 and December 2002. Mononuclear cells were isolated from samples at diagnosis by a Ficoll–Paque gradient as per the manufacturer's instructions (Amersham Pharmacia Biotech AB, Uppsala, Sweden). In all the cases, the diagnostic bone marrow sample contained blast cells in the ratio of at least 70%.

The median age at diagnosis in the study population as a whole was 14.5 years (range, 0.5–82 years). Of these patients, 92 were children (median age, 5 years; range, 0.5–14) and 91 presented adult ALL (median age, 35 years; range, 15–82). Diagnosis was established according to standard morphologic, cytochemical and cytogenetic criteria. Patients were studied at the time of initial diagnosis; were risk-stratified according to the therapeutic protocol used, which was always based on recognised prognostic features (including cytogenetics); and were entered in ALL protocols of the ‘*Programa para el estudio y tratamiento de las hemopatias malignas*’ (PETHEMA) Spanish study group. For statistical analyses, children were also grouped according to the National Cancer Institute (NCI) risk-classification criteria (Smith *et al*, 1996). The specific PETHEMA ALL treatment protocols in which these patients entered included ALL-89 (between 1989 and 1994; *n*=53) and ALL-93 (between 1994 and 2002; *n*=130). The design and results of these studies have been previously reported (Ortega, 1998; Ribera *et al*, 1998; Ortega *et al*, 2001; Ribera *et al*, 2002). In all, 64 patients relapsed. Overall, 29 patients received stem cell transplantation (10 autologous, 19 allogeneic) in the first (*n*=12) or second complete remission (CR) (*n*=17). There are 91 patients currently alive. Clinical characteristics of the patients are listed in Table 1

**Table 1:** Clinical characteristics and outcome of 183 ALL patients according to *Dkk-3* gene methylation status

**Feature**	**Nonmethylated (*n*=123), %**	**Methylated (*n*=60), %**	***P***
*Age*			NS
Younger than 15 years	48	52	
Older than 15 years	52	48	
Sex			
M/F	64/36	72/28	NS
			
*WBC*			NS
Below 50 × 10^9^ l^−1^	78	70	
Above 50 × 10^9^ l^−1^	22	30	
			
*FAB classification*			NS
L1	38	25	
L2	50	64	
L3	12	11	
			
*Blast lineage*			NS
B	88	64	
T	12	36	
			
*NCI risk groups*			NS
Standard	80	65	
Poor	20	35	
			
*Pethema risk groups*			NS
Standard	40	34	
Poor	60	66	
			
*Treatment*			NS
Pethema 89	25	27	
Pethema 93	75	73	
BMT	19	10	NS
			
*Best response*			NS
CR	90	92	
			
*Cytogenetic/molecular*			
*Abnormalities*			NS
BCR-ABL	17	14	
t(1;19)	4	2	
11q23	3	3	
c-Myc	6	8	
7q35–14q11	6	8	
Hyperdiploidy	9	5	
TEL-AML1	5	3	
Normal	46	52	
Others	3	3	
NT	1	2	
Relapse	34	57	0.009
Death	41	48	NS

Data are expressed as percentages; WBC=white bood count; FAB=French–American–British; NCI=National Cancer Institute; PETHEMA=Programa para el estudio y tratamiento de las hemopatias malignas; BMT=bone marrow transplantation; CR=complete remission.

. Some of these patients (*n*=105) were typed previously for methylation of the *Calcitonin* gene (Roman-Gomez *et al*, 2001).

### Quantitative real-time PCR (qrt-PCR) for *Dkk-3* expression

Total RNA was extracted from mononuclear marrow cells with Ultraspec (Biotecx, Houston, TX, USA) following the manufacturer's instructions. Reverse transcription was performed on 1 *μ*g total RNA, after heating at 70°C for 5 min, with random hexamers as reaction primer. The reaction was carried out at 42°C for 45 min in the presence of 12 U Avian Myeloblastosis virus reverse transcriptase (Boehringer-Mannhein, Germany). qrt-PCR was performed in a rapid fluorescent thermal cycler with three-colour fluorescence monitoring capability (LightCycler, Roche), using 1 *μ*l of cDNA in 20 *μ*l reaction volume with 0.4 *μ*mol l^−1^ each primer, and 2 *μ*l of 10 × LightCycler FastStar DNA Master SYBR Green I (Roche Molecular Biochemicals). The final Mg^2+^ concentration in the reaction mixture was adjusted to 3.5 mmol l^−1^. Primer set was specific for the *Dkk-3* gene (*Homo sapiens Dkk-3* mRNA, GeneBank: AB033421; sense, 5′-CTGTGTGTCTGGGGTCACTG-3′; antisense, 5′-GCTCTAGCTCCCAGGTGATG-3′). The following programme conditions were applied for qrt-PCR running: denaturation programme, consisting of one cycle at 95°C for 8 min; amplification programme, consisting of 45 cycles at 95°C for 5 s, 60°C for 10 s and 72°C for 15 s; melting programme, one cycle at 95°C for 0 s, 40°C for 60 s and 90°C for 0 s; and cooling programme, one cycle at 40°C for 60 s. The temperature transition rate was 20°C s^−1^, except in the melting programme, which was 0.4°C s^−1^ between 40 and 90°C.

Amplification of *Glyceraldehyde-3-phosphate dehydrogenase* (*GAPDH)* gene transcript was performed to assess RNA integrity and as reference gene (forward, 5′-TGAAGGTCGGAGTCAACGGATTTGGT-3′; reverse, 5′-CATGTGGGCCATGAGGTCCACCAC-3′). It was amplified in the same run and following the same procedure described above for *Dkk-3*. In order to reduce the variation between different assays and samples, a procedure based on the relative quantification of target genes *vs* their controls in relation to the reference gene was used. Calculations were automatically performed by LightCycler software (RealQuant, version 1.0, Roche). The normalised ratio was obtained from the next equation and expressed as percentage of the control:


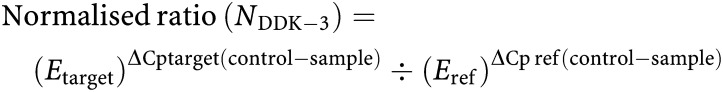


Efficiencies (*E*) of each gene were calculated from the slopes of crossover points (Cp) *vs* cDNA concentration plot, according to the formula *E*=10^(−1/slope)^. ΔCp corresponded to the difference between control Cp and sample Cp, either for the target or for the reference genes. The selected control was the bone marrow specimen from a healthy donor. It was considered as 100% expression. Occasionally, equal amounts of PCR products were separated on a 2% agarose gel, stained with ethidium bromide, and visualised under UV light.

### Methylation-specific PCR (MSP) of *Dkk-3* promoter

Analysis of the *Dkk-3* gene (GeneBank: AB045205, *Homo sapiens Dkk-3* gene) has revealed that *Dkk-3* promoter was CpG-rich, showing >60% C+G content and an observed-overexpected CpG frequency of >0.6, satisfying the criteria for a CpG island. Aberrant promoter methylation of *Dkk-3* gene was determined by method of MSP as previously reported (Herman *et al*, 1996). MSP distinguishes unmethylated alleles of a given gene based on DNA sequence alterations after bisulphite treatment of DNA, which converts unmethylated but not methylated cytosines to uracils. Subsequent PCR using primers specific to sequences corresponding to either methylated or unmethylated DNA sequences was then performed. Primer sequences of *Dkk-3* for the unmethylated reaction were forward (5′-TTTTGGTTTTTTTTTGTTTTTGGG-3′) and reverse (5′-CCAAACCACTACATCTCCACT-3′), which amplify a 179-bp product. Primer sequences for the methylated reaction were forward (5′-CGGTTTTTTTTCGTTTTCGGG-3′) and reverse (5′-CAAACCGCTACATCTCCGCT-3′), which amplify a 175-bp product. Briefly, 1 *μ*g of genomic DNA were denatured by treatment with NaOH and modified by sodium bisulphite. DNA samples were purified using Wizard DNA purification resin (Promega Corp., Madison, WI, USA), treated with NaOH, precipitated with ethanol, and resuspended in 20 *μ*l of water. A volume of 2 *μ*l of modified DNAs were PCR amplified in a total volume of 50 *μ*l. ‘Hot start’ PCR was performed for 35 cycles consisting of denaturation at 95°C for 1 min, annealing at 60°C for 1 min, and extension at 72°C for 1 min, followed by a final 7-min extension for all primer sets. DNA from mononuclear marrow cells (*n*=20) from healthy donors were used as negative controls for methylation-specific assays. Human male genomic DNA universally methylated for all genes (Intergen Company, Purchase, NY, USA) was used as a positive control for methylated alleles. Water blanks were included with each assay. PCR products were visualised on 2% agarose gels stained with ethidium bromide. Results were confirmed by repeating bisulphite treatment and MSP assays for all samples. The sensitivity of this MSP was established by using totally methylated, positive control DNA serially diluted by normal lymphocyte DNA. MSPs with 1 : 10, 1 : 100, and 1 : 1000 diluted positive control DNA produced detectable methylated bands (data not shown).

### Western blot analysis of Dkk-3 protein expression

For immunoblotting, proteins were extracted from B-lineage ALL-derived MY cell line and MY cell line treated with 1, 2, 4 or 6 *μ*M of 5-Aza-2′-deoxycytidine during 4 days. Cells from MY and treated MY cell lines were washed in PBS and lysed in Triton lysis buffer (1% Triton X-100, 50 mM Tris-HCl pH 8, 150 mM NaCl, 1 mM PMSF and 1% aprotinin). The supernatant was collected by centrifugation at 15 000 ***g***, 20 min, 4°C, and the protein concentration was determined using BCA protein assay kit (Pierce, Rockford, IL, USA). Cell lysates (containing 50 *μ*g of proteins) were size-fractionated by 10% SDS–PAGE and electrotransferred to nitrocellulose membranes. The membranes were blocked with I-Block™ (Tropix, Bedford, MA, USA) and incubated for 2 h with goat polyclonal antibody against Dkk-3 (Santacruz Biotechnology, Santa Cruz, CA, USA) diluted 1 : 750 and then with donkey anti-goat antibody conjugated to alkaline phosphatase (Promega, Madison, WI, USA) diluted 1 : 15 000 for 45 min. As loading control, the membranes were incubated for 1 h with mouse monoclonal antibody against *β*-tubulin diluted 1 : 1000 and then with goat anti-mouse antibody conjugated to alkaline phosphatase (Sigma, Steinheim, Germany) diluted 1 : 10 000 for 45 min. Finally, bound antibodies were detected by a chemiluminescence system (Tropix, Bedford, MA, USA). For detection of secreted proteins, 20 *μ*l of filtered, dialysed, conditioned media, collected after 48 h of incubation with MY and treated MY cell lines, was analysed by Western blotting.

### Statistical analysis

*P*-values for comparisons of continuous variables between groups of patients were two-tailed and based on the Wilcoxon rank-sum test. *P*-values for dichotomous variables were based on the Fisher exact test. The remaining *P*-values were based on the Pearson χ^2^-test. Overall survival (OS) was measured from the day of diagnosis until death from any cause and was only censored for patients known to be alive at last contact. Disease-free survival (DFS) was measured from the day that complete response (CR) was established until either relapse or death without relapse, and it was only censored for patients who were alive without evidence of relapse at the last follow-up. Distributions of OS and DFS curves were estimated by the method of Kaplan and Meier with 95% confidence intervals calculated using Greenwood's formula. Comparisons of OS or DFS between groups were based on the log-rank test. Comparisons adjusted for significant prognostic factors were based on Cox regression models and hazard regression models. All relapse and survival data were updated on 31 December 2003 and all follow-up data were censored at this point.

## RESULTS

### *Dkk-3* expression is downregulated in ALL samples and T/B-precursor ALL cell lines

**Figure 1:**
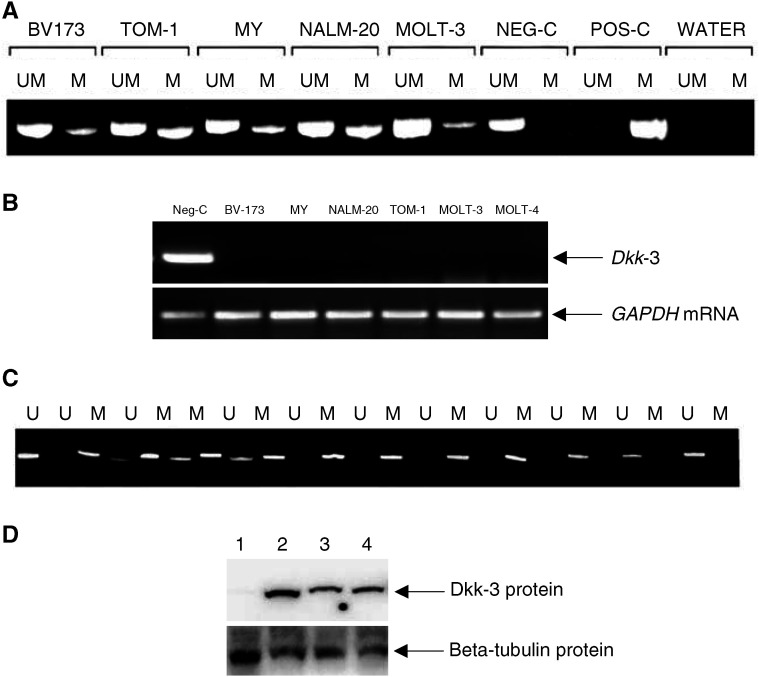
Methylation status and expression levels of *Dkk-3* in ALL cell lines and patients. (**A**) MSP analysis of CpG island within *Dkk-3 promoter* in five ALL cell lines. Pos-C indicates methylated control; Neg-C indicates unmethylated control (marrow mononuclear cells from a healthy donor); UM=unmethylated alleles; M=methylated alleles. Promoter hypermethylation is observed in all cell lines. (**B**) RT–PCR analysis with the *Dkk-3* and *GAPDH* (as control for mRNA integrity) primers. Neg-C indicates unmethylated control. Lack of *Dkk-3* expression in observed in all cell lines. (**C**) MSP analysis of CpG island within *Dkk-3 promoter* in 12 ALL patients. (**D**) Analysis of Dkk-3 protein expression by Western blot. The levels of beta-tubulin were also analysed as loading control. 1: ALL-derived MY cell line; 2–4: MY cell line treated with 2, 4 or 6 mM of 5-Aza-2′-deoxycytidine. The demethylating agent restores Dkk-3 expression.

To determine the cutoff point for altered *Dkk-3* expression in ALL samples, the *N*_DDK-3_ value for *Dkk-3* was determined in 20 bone marrow samples from healthy donors. *N*_DDK-3_ values fell between 107 and 86% (mean: 96.5±5.6%). A *N*_DDK-3_ value below 68.5% (determined as the mean±5 s.d.) was chosen to define underexpression of *Dkk-3* in ALL RNA samples. No *Dkk-3* expression was observed in T- or B-precursor ALL cell lines (Figure 1B

). In addition, we found a strong reduction of *Dkk-3* mRNA in 41% of diagnostic ALL samples (*N*_DDK-3_: mean, 23%; range, 47–0%). No overexpression of *Dkk-3* (*N*_DDK-3_ above 107%) was observed in ALL primary tumours.

### Methylation of *Dkk-3* promoter is associated with reduced mRNA expression

To investigate a mechanism for the reduced expression of the *Dkk-3* gene, we analysed *Dkk-3* promoter, which is known to be methylated in cancer cell lines lacking *Dkk-3* expression (Kobayashi *et al*, 2002). Promoter of the *Dkk-3* gene has a typical CpG island showing >60% C+G content and an observed-overexpected CpG frequency of >0.6. By MSP, CpG island of the *Dkk-3* promoter was revealed to be highly methylated in all ALL cell lines (Figure 1A) and in 60 of 183 (33%) samples from patients with ALL (Figure 1C), which was clearly in contrast to no *Dkk-3* methylation in normal cells.

All 60 methylated ALLs (100%) showed decreased *Dkk-3* expression; in contrast, decreased *Dkk-3* expression was detected in only 10 of 123 ALLs with unmethylated pattern (8%). This result indicated that CpG methylation within *Dkk-3* promoter strongly correlated with decreased constitutive expression of *Dkk-3* in ALL cells (*P*<0.0001).

Exposure to different concentration of the demethylating agent 5-Aza-2′-deoxycytidine restored, in both cell lysate and culture supernatant, the expression of Dkk-3 protein in the MY ALL cell line indicating that hypermethylation is a major mechanism by which Dkk-3 expression is silenced in ALL cells (Figure 1D).

Dkk-3 methylation, clinical presentation and outcome: Dkk-3 methylation was detected at diagnosis in 33% (60 out of 183) of patients with adult or childhood ALL of all the FAB subtypes. As shown in Table 1, aberrant Dkk-3 methylation was similarly observed in adults (30 out of 91, 33%) and children (30 out of 92, 32%). Clinical and laboratory features did not differ significantly between methylated and normal patients. Poor-risk cytogenetics or molecular events, risk groups according to both NCI or PETHEMA classifications, good risk features (hyperdiploidy and TEL-AML1 fusion), type of PETHEMA protocol administered and number of patients who received stem cell transplantation were similarly distributed between the two groups defined by Dkk-3 methylation. Separate analysis of adult and childhood ALL patients gave the same results as the global series. The presence of methylation in the Calcitonin gene was determined previously for 105 of these cases (Roman-Gomez *et al*, 2001). There was a significant concordance between Dkk-3 methylation and the presence of Calcitonin methylation; methylation was present in 30 out of 42 (71.4%) Calcitonin-methylated tumours *vs* 13 out of 63 (20.6%) Calcitonin nonmethylated tumours (*P*=0.0001).

Table 1 details the relapse history, CR rates and mortality for patients who exhibited Dkk-3 methylation and the equivalent data for patients with normal *Dkk-3* gene. CR rates of patients with normal and methylated *Dkk-3* gene were 90 and 92%, respectively, accounting for 91% of the overall CR rate. This suggests that methylation of the *Dkk-3* gene did not correlate with response to remission induction therapy. However, methylated patients had a higher relapse rate (57.7 *vs* 34.7%, *P*=0.009) than nonmethylated patients. Similar results were obtained in the separate analyses of children (relapse rate: 46 *vs* 25%, *P*=0.04) and adults (relapse rate: 71 *vs* 45%, *P*=0.03).

**Figure 2:**
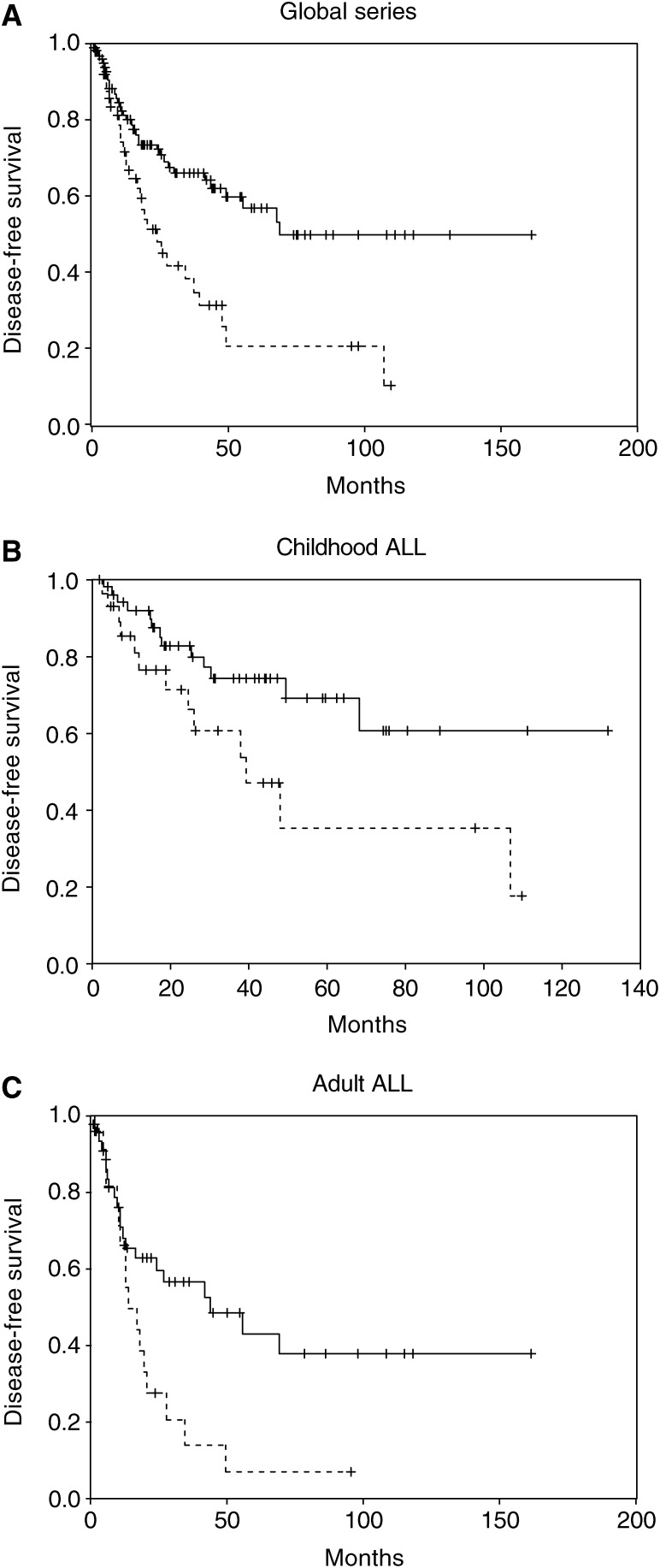
Kaplan–Meier survivor function for ALL patients. DFS curves for ALL patients enrolled in this study according to methylation status of the *Dkk-3* gene. Solid lines, unmethylated patients; dashed lines, methylated patients.

We analysed the DFS among patients who achieved CR according to Dkk-3 methylation. Estimated DFS rates at 10 years were 49.8 and 10.5% for normal and methylated patients, respectively (*P*=0.001) (Figure 2A

). Among unmethylated children, the 10 years DFS was 60.6% contrasting with 17.6% for hypermethylated children (*P*=0.03, Figure 2B). Among adult ALL patients, the 9-years DFS was 6.8% for methylated cases and 37.7% for normal cases (*P*=0.01, Figure 2C). The actuarial OS calculated for all leukaemic patients was 45.6 and 15.1% at 11 years for cases with normal and hypermethylated *Dkk-3* gene, respectively (*P*=0.09).

A multivariate analysis of potential prognostic factors (including the type of PETHEMA protocol applied) demonstrated that methylation of the *Dkk-3* gene was an independent prognostic factor in predicting DFS in the global series and also in both, childhood and adult ALL (Table 2

**Table 2:** Multivariate Cox model for disease-free survival

	**Univariate analysis**	**Multivariate analysis**
**Feature**	***P*-value**	***P*-value**
Global series		
Dkk-3 hypermethylation	0.0004	0.0009
WBC>50 000 mm^3^	<0.0001	0.01
BCR-ABL positivity	0.02	0.01
T phenotype	0.007	0.05
Age>15 years	0.05	—
Pethema poor risk	0.05	—
		
Childhood ALL		
Dkk-3 hypermethylation	0.003	0.003
T phenotype	0.05	0.04
WBC>50 000 mm^3^	0.02	—
		
Adult ALL		
WBC>50 000 mm^3^	0.0001	0.0006
Dkk-3 hypermethylation	0.05	0.05
BCR-ABL positivity	0.009	—

).

## DISCUSSION

Mammalian cells have a finite replicative potential *in vitro* and *in vivo* referred to as the Hayflick limit (Hayflick, 1997). Upon reaching this limit, cells cease to divide and undergo genetic, biochemical and morphological changes indicative of ageing, a state referred to as cellular senescence. As these cells do not proliferate, it has been suggested that cellular senescence is a major barrier against cancerous transformation. The ability to overcome senescence and obtain a limitless replicative potential (termed immortalisation), is one of the prerequisites for tumour transformation (Hanahan and Weinberg, 2000). If downregulation of a gene can lead to the immortalisation of cells, the gene may be a candidate of tumour suppressor gene. Recently, a mortalisation-related gene was isolated by the representational difference analysis system with an immortalised cell line and the normal counterpart (Tsuji *et al*, 2000). A search of this gene revealed that it was identical with the human *Dkk-3* gene, an antagonist of the Wnt oncogenic pathway. More recently, it has been reported that *Dkk-3* expression was reduced by methylation in several cancer cell lines and primary lung cancers (Kobayashi *et al*, 2002).

*Dkk-3* gene could be a good candidate for epigenetic inactivation in ALL. It is located near the Calcitonin gene, a gene known to be methylated in ALL patients with unfavourable prognosis (Roman-Gomez *et al*, 2001). However, this gene is generally considered not to be causally related to the malignant process, suggesting that methylation status of the Calcitonin gene region in ALL lymphoblasts may well correspond to that of its neighbouring *Dkk-3* gene. In this study, we have identified the *Dkk-3* gene as a target gene for methylation and silencing in ALL. *Dkk-3* locus methylation was detected in six ALL-derived cell lines and in 33% of tumours at diagnosis, indicating that inactivation of the *Dkk-3* gene is a frequent and early event in the process of tumorigenesis in this disease. Methylation of the *Dkk-3* promoter region was associated with loss of *Dkk-3* gene expression in neoplastic cells and this expression was restored after exposure to the demethylating agent 5-AzaC, indicating that hypermethylation is a major mechanism by which *Dkk-3* expression is silenced in ALL cells. Moreover, a significant concordance between Dkk-3 methylation and Calcitonin methylation was observed, suggesting that Calcitonin methylation is a surrogate for the methylation of the *Dkk-3* gene.

What is the functional significance underlying the methylation-mediated transcriptional loss of *Dkk-3* in ALL? During replicative senescence altered expression of genes that are involved in cell cycle control and oncogenesis is well documented. A senescence-like arrest can be induced by ectopic expression of *p15*^*INK4b*^, *p16*^*INK4a*^, *p21*^*CIP*^ or by DNA-damaging agents that activate *p53* (Serrano *et al*, 1997; Stein *et al*, 1999). These findings are consistent with a model in which the concurrent induction of a mitogenic stimulus with a forced G_1_ arrest may be sufficient to induce a senescent phenotype. Senescence, much like apoptosis, represents a major barrier to tumorigenesis and it can be achieved by shortened telomeres or conflicting growth signals that forces aberrant cells into a G_0_-like state (Hanahan and Weinberg, 2000). The genetic changes associated with immortalisation, namely loss of *p16*, *p15*, *p21* and *p53*, are among the most common known cancer-related changes, and these genes are frequently downregulated by methylation in ALL (Garcia-Manero *et al*, 2002; Roman-Gomez *et al*, 2002; Agirre *et al*, 2003). *Dkk-3* could function in a similar way, confirming that epigenetic inactivation of genes involved in senescence regulation plays a major role in the pathogenesis of leukaemia. In addition, *Dkk-3* could contribute to leukaemogenesis by means of its ability to antagonise Wnt proteins. Wnt pathway plays a crucial role in haematopoietic differentiation (Brandon *et al*, 2000); and activation of a ‘canonical’ Wnt pathway is associated with several types of human cancers, including B-cell malignancies (Qiang *et al*, 2003). The ‘transforming activity’ of Wnt is ascribed to the beta-catenin/signalling pathway, which appears to be specifically activated by Wnt7A protein (Sheldahl *et al*, 1999). Interestingly, *Dkk-3* negatively modulates Wnt7A signalling (Caricasole *et al*, 2003). Therefore, it can be speculated that lack of *Dkk-3* expression favours the activation of the most powerful Wnt protein related to the development of cancer.

These findings, together with the prognostic impact of *Dkk-3* inactivation in ALL, the general expression of *Dkk-3* in normal blood cells, the emerging role of the *Dkk-3* gene in human cancer and the observation that transfection of human osteosarcoma Saos-2 cells with *Dkk-3* entails a decrease in the proliferative activity are supportive of *Dkk-3* inactivation contributing directly to the clinical behaviour of ALL, at least in a subgroup of patients.

In conclusion, our results strongly indicate that the silencing of *Dkk-3* expression by aberrant promoter methylation occurs at an early stage of ALL pathogenesis and plays a role in the outcome of the disease.
